# Through-the-Wall Localization of a Moving Target by Two Independent Ultra Wideband (UWB) Radar Systems

**DOI:** 10.3390/s130911969

**Published:** 2013-09-09

**Authors:** Dušan Kocur, Mária Švecová, Jana Rovňáková

**Affiliations:** Department of Electronics and Multimedia Communications, Technical University of Košice, Park Komenského 13, Košice 04120, Slovakia; E-Mail: svecova.maria@gmail.com

**Keywords:** collaborative localization, direct calculation method, ellipse intersections, radar signal processing, target localization, Taylor series method, UWB radar

## Abstract

In the case of through-the-wall localization of moving targets by ultra wideband (UWB) radars, there are applications in which handheld sensors equipped only with one transmitting and two receiving antennas are applied. Sometimes, the radar using such a small antenna array is not able to localize the target with the required accuracy. With a view to improve through-the-wall target localization, cooperative positioning based on a fusion of data retrieved from two independent radar systems can be used. In this paper, the novel method of the cooperative localization referred to as joining intersections of the ellipses is introduced. This method is based on a geometrical interpretation of target localization where the target position is estimated using a properly created cluster of the ellipse intersections representing potential positions of the target. The performance of the proposed method is compared with the direct calculation method and two alternative methods of cooperative localization using data obtained by measurements with the M-sequence UWB radars. The direct calculation method is applied for the target localization by particular radar systems. As alternative methods of cooperative localization, the arithmetic average of the target coordinates estimated by two single independent UWB radars and the Taylor series method is considered.

## 1. Introduction

Localization capability is becoming one of the most attractive features of a wireless sensor network. Ultra wideband (UWB) radar systems as a special kind of wireless sensor network allow one to detect and track authorized or unauthorized moving targets with an advantage in critical environments or under hindered conditions. This results from the fact that UWB radars operating in the frequency range up to 4 GHz are characteristic of a good penetration of emitted electromagnetic waves through various materials, such as wood, brick, concrete, plastic, rock, ground, snow, *etc*. Therefore, such radars are able to detect moving person by measuring changes in the impulse response of complex monitored environments [1]. There are a number of applications where UWB radar systems can be very helpful, e.g., for through-the-wall detection and localization of persons during security operations or fire, detection of people trapped in an avalanche or an earthquake, monitoring of critical infrastructures (reservoirs, power plants, *etc*.) or through-the-wall imaging of building layouts and interiors [2].

In these applications, handheld sensors for through-the-wall monitoring are applied by security forces directly to the place of operation. Here, the monitoring results should be available for the radar operator immediately. It is also expected that the operator can change the sensor location permanently. It follows from the outlined requirements that the handheld radar systems have to operate in a stand-alone mode and to be small-sized and light-weight. Therefore, they usually use only a small antenna array necessary for motion detection and basic spatial positioning of the targets.

It is well-known that the ability to detect moving targets and target positioning precision depend inter alia on the number of the radar antennas and their size, types and spatial layout. However, in the case of handheld sensors, the radar system performance improvement based on the application of a complex antenna array is naturally limited. The alternate approach on how to improve the detection and positioning is to use at the same time two or more handheld sensors for monitoring the same area. Here, diversity is the keyword behind the outlined idea. By spacing the particular radar systems, including their transmitting and receiving antenna elements, in such a way that the target angular spread is manifested, the sensor network can exploit the spatial diversity of target scatters and mitigate to environment complexity, opening the way to a variety of new techniques that can improve radar performance [3]. Then, the proposed approach is similar to that of the statistical multiple-input multiple-output (MIMO) radar concept, capitalizing on the angular diversity principle. Incorporating the idea of angular diversity application in our concept of target localization by two independent radar systems, improvement of the detection reliability and localization accuracy can be reached by a cooperative localization of the targets based on a fusion of data obtained from the particular handheld sensors.

Motivated by the outlined scenarios of the UWB radar applications, we will focus on through-the-wall localization and tracking of a moving person by two independent UWB radar systems in this paper (hereinafter, the basic scenario). In the mentioned scenario, the handheld M-sequence UWB radars equipped with one transmitting and two receiving antennas will be used as UWB sensors [4,5].

Moving target localization and tracking by two independent UWB radar systems is a complex procedure that includes signal processing phases, such as background subtraction, detection, time-of-arrival (TOA) estimation, wall effect compensation, localization and tracking. The significance of the particular phases has been explained, e.g., in [6–8]. With the exception of the localization phase, other phases of radar signal processing are independent of the number of UWB radar systems applied for target tracking. Therefore, in this paper, we will focus on the localization task solution. Here, the target coordinates in a defined coordinate system are the localization phase result.

In the case of the single radar system equipped with one transmitting and two receiving antennas, the direct calculation (DC) method is the basic method of target localization [7,9]. On the other hand, for the purpose of target localization by the UWB sensor networks with a general number of nodes, a huge number of localization methods based on TOA measurements can be used [10]. Here, the simple arithmetic average of the target coordinates estimated by the particular nodes (MEAN), the least-squares method [11–13], the constrained weighted least-squares method [14,15], the spherical-interpolation method [16,17], the Taylor series method [9,12,18] and various methods based on optimization principles [11,19] can be listed.

In addition to these papers, several interesting contributions (e.g., [20–23]) devoted predominantly to the target localization by UWB sensor networks based on TOA estimation have been published recently.

In [20], a framework for design and analysis of UWB monostatic radar networks for passive localization and navigation has been presented. The framework accounts for the network setting, environment propagation, TOA estimation techniques and Bayesian navigation algorithms. Navigation techniques based on the particle filter algorithm and mobility models have been compared in terms of navigation error outage for a case study in an indoor environment with both line-of-sight and obstructed line-of-sight conditions.

Target localization by a multistatic UWB radar system has been studied in [21]. In the considered system, at each scanning, each receiving node calculates a soft image of the surveillance area based on the target-scattered UWB pulses. All images are then transferred to a fusion node, where the decision about target presence or absence is taken, and subsequently, the target coordinates are estimated. Optimum decision metrics and likelihood tests are developed, together with approximated metrics, reducing the complexity of the detection block. Moreover, it has been explained how they may be combined with a tracking approach based on Bayesian filtering to improve the localization accuracy.

Another interesting approach to target localization by UWB sensor networks represented by multistatic UWB radar has been introduced in [22]. In this contribution, a novel, two-step estimation (TSE) algorithm for the object localization has been developed. It has been shown in [22] that the TSE algorithm achieves the Cramer-Rao lower bound when the TOA measurements are subjected to small Gaussian-distributed errors, which is verified by analytical and simulation results.

The mentioned localization methods applied for scenarios with a general number of nodes are essentially based on an appropriate solution of the overdetermined set of nonlinear equations and/or a proper optimization task solution. The particular equations are created by means of the known coordinates of the radar antennas and TOA estimations corresponding to the target to be localized. Because of the optimization approach application, the performance properties of these localization methods depend strongly on the number of the nodes. The more nodes are used, the better the performance of the localization methods will be obtained. On the other hand, if the number of the equations is small, the target localization precision is very sensitive to TOA estimation accuracy. In this case, even a single TOA estimated with a large error results in a large error of the target position estimation. Unfortunately, this scenario is just a typical one for through-the-wall localization of the target by means of two independent radar systems. In this case, only four nonlinear equations are available for the target localization problem description [24]. Besides, the monitored area for through-the-wall localization is complex, and hence, the particular TOA can be frequently estimated with a large error. These short analyses indicate that the application of modified localization methods developed originally for a general number of nodes (radars) does not have to provide a meaningful improvement of the target positioning accuracy and the robust performance for the target localization by two independent radar systems.

The alternative solution of the target localization problem by two independent radar systems referred to as joining intersections of the ellipses (JIEM) has been originally proposed in [25]. The main idea of JIEM consists in the finding of all the possible positions of the target in the monitored area by using DC. Then, a cluster of the target positions having the potential to be closest to the true target position is created by the novel sophisticated decision algorithm. Finally, the target coordinates are estimated as the average values of the coordinates of all possible positions of the target included in the mentioned cluster. It follows from the outlined principle of JIEM that JIEM is not based on an optimization principle, but on the selection of the best candidates for the true target position. Therefore, it can be expected that JIEM could be more robust to TOA estimation error for the discussed scenario than the methods using the optimization principle.

In this paper, JIEM will be derived in detail. In order to compare the performance of JIEM and a method based on the direct solution of a nonlinear overdetermined equation set, a new modification of the Taylor-Series method (TSM) adapted for the basic scenario problem will be introduced also in our contribution. Then, the performance of the DC (applied for each radar system independently), MEAN, TSM and JIEM will be compared based on the processing of the signals obtained by through-the-wall measurement with two independent M-sequence UWB radar systems. For raw radar data processing, the complex UWB radar signal procedure proposed in [6] will be used. The obtained results will show very clearly that JIEM is able to reach, at the cost of its relatively high computational complexity, more precise and robust estimation of the target trajectory than that of DC, MEAN or TSM. In contrast to our previous work [25], this contribution contains not only JIEM derivation, but also MEAN and TSM introduction, the extended comparison of single sensor application (*i.e.*, DC method application), MEAN and TSM with JIEM, including not only the target trajectory and track estimation, as in [25], but also, the time evolution of target localization errors and its analyses, deeper analyses of the JIEM properties and the discussion of the further possible extension of JIEM for some other scenarios.

To fulfill the outlined intention, our paper will have the following structure. In Section 2, the problem statement for the target localization by means of two independent UWB radar systems considered in this paper will be given. In Section 3, the particular phases of radar signal processing will be outlined. Section 4 is the core of our contribution. In this section, TSM and JIEM will be introduced. Subsequently, the performance of DC, MEAN and the introduced localization methods will be illustrated, compared and discussed in Section 5. Finally, conclusions and final remarks to this contribution are made in Section 6.

## 2. Problem Statement

**Figure 1.**
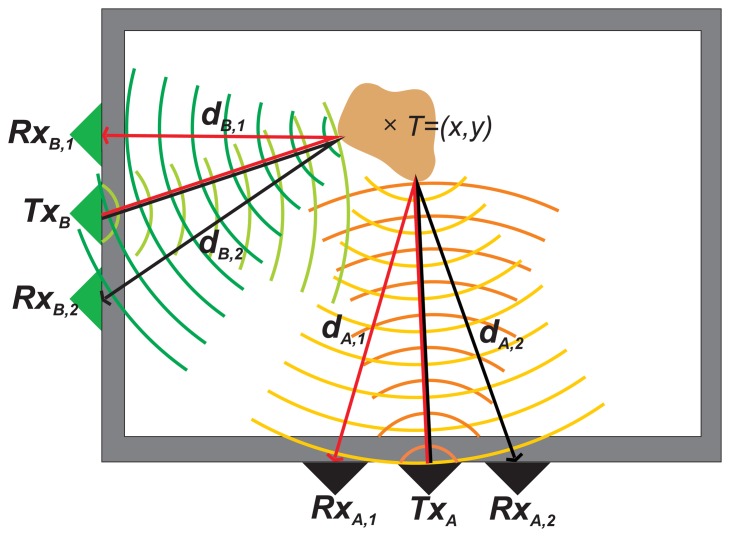
The target localization by two ultra wideband (UWB) radar systems. The basic scenario.

Let us consider the fundamental scenario of through-the-wall localization of a moving target by means of two UWB radar systems, denoted as the radar system A (RS_A_) and the radar system B (RS_B_) (Figure 1).

Here, every radar system is equipped with one transmitting and two receiving antennas. In the scenario, it is assumed that the antenna positions are known, and their coordinates are given as follows:

- coordinates of the transmitting antenna of the RS_A_(*Tx_A_*): *Tx_A_* = (*x_A,t_,y_A,t_*),
- coordinates of the first receiving antenna of the RS_A_: (*Rx_A_*_,1_): *Rx_A_*_,1_ = (x*_A_*_,1_, *y_A_*_,1_),
- coordinates of the second receiving antenna of the RS_A_(*Rx_A_*_,2_): *Rx_A_*_ ,2_ = (*x_A_*_,2_,*y_A_*_ ,2_),
- coordinates of the transmitting antenna of the RS_B_ (* Tx_B_*): *Tx_B_* = (*x_B,t_*, *y_B,t_*),
- coordinates of the first receiving antenna of the RS_B_(*Rx_B_*_,1_): *Rx_B_*_,1_ = (x*_B_*_,1_, *y_B_*_,1_),
- coordinates of the second receiving antenna of the RS_B_(*Rx_B_*_,2_): *Rx_B_*_,2_ = (*x_B_*_,2_, *y_B_*_,2_).

Raw radar signals retrieved from the particular radar systems can be interpreted as a set of impulse responses of the surroundings through which the electromagnetic waves emitted by the radar are propagated. They are aligned to each other, creating a 2D picture called a radargram, where the vertical axis is related to the propagation time (*t*) of the impulse response and the horizontal axis is related to the observation time (*τ*) [4,6].

Hereinafter, we will assume that the radar systems applied for target tracking are synchronized in such a way that both radar devices are controlled approximately by the same system clock. Therefore, we can assume that the radargrams obtained by the measurements by all four receiving antennas have the same propagation and observation time axes and that their radargram samples are taken in the same time instants. The other kind of radar system synchronization is not assumed.

Taking into account the above-mentioned assumption, the problem to be solved within our paper is to estimate the target trajectory based on processing of raw radar signals retrieved from two independent radar systems. The solution of that problem will consist of two stages. Within the former stage, TOA corresponding to the target for each pair of transmitting and receiving antennas of the same radar system will be estimated. For that purpose, the UWB radar signal processing procedure described in the next section will be applied. The latter stage of the target localization will consist in the target coordinate estimation based on the fusion of the data (*i.e.*, TOA) retrieved from radar systems RS_A_ and RS_B_. For that purpose, TSM and JIEM will be proposed in Section 4. The further improvement of target positioning accuracy will be reached by target tracking. The tracking algorithms applied in our paper will be mentioned in Section 3.

## 3. UWB Radar Signal Processing Procedure

In the case of UWB radar signal processing for through-the-wall localization of moving persons, target positioning is a complex procedure that includes such signal processing phases as background subtraction, target detection, TOA estimation, wall effect compensation, target localization and tracking. The particular phases are implemented using appropriate methods of signal processing. In the following parts of this section, we would like to provide the reader with a short outline of the mentioned procedure. It should help for readers to see clearly the connections of the target localization methods developed and discussed in this paper with their applications for through-the-wall localization of moving persons by UWB radar systems. Ea intentione, the significance of the particular phases of the mentioned procedure will be outlined, and the lists of signal processing methods that are most frequently used within the particular phases will be given. Because of the complexity of the discussed procedure of moving target positioning, its detailed description is beyond this paper, and hence, it will not be presented here. The reader can find its comprehensive description, especially, in [6,26,27].

### 3.1. Background Subtraction

The analysis of raw radar data has shown that it is impossible to directly identify any moving targets in the obtained radargrams. This comes from the fact that the components of the impulse responses represented by the target echo are much smaller than those of the signals reflected by the front wall or large or metal static objects or signals representing the cross-talk between transmitting and receiving antennas. In order to detect a moving target, the ratio of signals scattered by a target (*i.e.*, nonstationary components of received signals) to noise and clutter (*i.e.*, stationary components of received signals) has to be increased. For that purpose, background subtraction methods can be used. They help to reject, especially, stationary and correlated clutter, such as antenna coupling, impedance mismatch response and ambient static clutter, and in such a way, they allow one to detect a moving target echo.

It has been shown that the signal processing methods, such as basic averaging (mean, median) [28], exponential averaging [29], adaptive exponential averaging [29], adaptive estimation of Gaussian background [30], Gaussian mixture method [31], moving target detection by finite impulse response (FIR) and infinite impulse response (IIR) filtering [32,33], prediction [34], principal component analysis [35], etc., can be used for background subtraction. These methods differ in relation to assumptions concerning the clutter properties, as well as by their computational complexity and convenience for online signal processing. Because of a good performance, high robustness and low computational complexity, the method of exponential averaging belongs to the most popular and often used methods of background subtraction.

### 3.2. Detection

Detection is the next phase of the radar signal processing procedure, which comes after the background subtraction. Detection methods analyze the radargram with the subtracted background and reach the decision of whether a signal scattered by a moving target is present or absent in the analyzed impulse response.

The detector output corresponding to the receiving antenna in the observation time instant, *τ_k_*, is represented by a binary sequence, *h_d_* (*t*, *τ_k_*) [26]. The non-zero sample of the detector output in the propagation time instant, *t_j_*, indicates that there is an echo, due to the target or a false alarm in the observation time instant, *τ_k_*, whereas the distance between the transmitting antenna, target and receiving antenna is *cTOA_kj_*, where *c* is the velocity of the propagation of the electromagnetic waves emitted by the radar and *TOA_kj_* = *t_j_* is the time of arrival corresponding to the target detected in the observation time instant, *τ_k_*, and propagation time instant, *t_j_*.

The detailed structure of a detector depends on the selected strategy and optimization criteria of detection [36,37]. For the purpose of the moving target detection by using UWB radars, detectors with fixed threshold, (N,k) detectors [37], inter-period correlation processor (IPCP) detectors [38] and constant false alarm rate (CFAR) detectors [39] have been proposed.

CFAR detectors can be especially assigned between detectors capable of providing a good and robust performance for through-the-wall detection of moving targets by the UWB radar system. They are based on the Neymann-Person optimum criterion, providing the maximum probability of detection for a given false alarm rate. There are a number of varieties of CFAR detectors [40]. For example, the CFAR detector adapted especially for UWB radar signal processing has been introduced in [41]. In spite of its simple structure and the assumption of the Gaussian model of clutter, it has proven to have very good and robust performance for many scenarios of through-the-wall detection of moving targets.

### 3.3. TOA Estimation

If a target is represented by only one non-zero sample of the detector output, then the target is referred to as a simple target. However in the case of the scenario analyzed in this contribution, the radar range resolution is finer than the physical dimensions of the target. This results in the detector output, due to such a target, usually not being expressed by only one non-zero sample of *h_d_*(*t*, *τ_k_*), but by the whole set of the non-zero samples of the detector output. In this case, the target is referred to as the distributed target [26]. Since several different TOA correspond to the same distributed target, the detector output for a distributed target is very complex, and the task for the distributed target localization is more complicated than that for a simple target. The basic idea of the distributed target localization applied in this paper consists of a substitution of the set of TOA corresponding to the same target with only one non-zero properly estimated TOA referred to as the TOA of the distributed target. Then, the TOA corresponding to a distributed target will be expressed by only one instant of the propagation time, *t_k_*. Consequently, by using TOA defined in such a way, the distributed target position can be determined by using the same approach as for a simple target. In order to improve the readability of the paper, the term TOA will be used only for the TOA of the distributed target throughout this paper.

The TOA estimation is quite complex, and therefore, it is not fully described in this paper. An algorithm of this kind can be found, e.g., in [42]. This algorithm provides not only TOA estimation for the distributed target, but also the association of the data received from two receiving channels and a deghosting operation essential for multiple target detection and tracking scenarios. Another approach for TOA estimation suitable for the considered application is described in [20,43]. In [43], two methods of TOA estimation have been proposed. The former method is based on the application of a CFAR detector. The latter one exploits the maximum probability of the detection method, determining the TOA estimation according to a comparison of the detection probabilities of a number of different possible TOA estimations.

On the other hand, the low complexity TOA estimation for UWB systems based on model selection by information theoretic criteria has been developed in [20]. The resulting TOA estimation algorithms do not use thresholds and do not require any information about the channel or the noise power level. These blind, universal TOA estimators show, for completely unknown multipath channels and in the presence of noise with unknown power, excellent performance when compared with ideal genie-aided schemes.

### 3.4. Wall Effect Compensation

The propagation of electromagnetic waves through-the-wall results in a delayed time of signals reflected by targets moving behind the wall, which means that TOA estimated by the previous phase of the radar signal processing are time shifted, because of the wall presence. Their correction can be achieved by the subtraction of the mentioned delay time, whereby its estimation is the task of the wall effect compensation phase. The method referred to as the target trace correction of the second kind [44] provides promising results in this area. For its utilization, the wall parameters, such as permittivity, permeability and thickness of the wall, have to be known in advance. The mentioned parameters can be estimated very efficiently, e.g., by UWB radar using the method described in [45]. Seeing that in the basic scenario, the walls are thin with small relative permittivity and taking into account the size of the target, the wall effect can be treated as negligible in that case. Therefore the wall effect compensation phase will be omitted from the processing of signals obtained in the basic scenario.

### 3.5. Localization

The aim of the target localization phase is to determine the target coordinates in a defined coordinate system. The target positions estimated in the consecutive observation time instants create a target trajectory. The analyses of the target localization by M-sequence UWB radar equipped with one transmitting and two receiving antennas based on DC presented in [46] has confirmed that the distance between transmitting and receiving antennas, TOA estimation error and the target position in the monitored area are the key factors determining the localization error. It has been shown in [46] that the localization error can take on values from zero up to several meters, even for very simple scenarios. These conclusions indicate that that cooperative localization of the target based on the sensor network could be the key solution for the improvement of the accuracy of the target localization. As the phase of the target localization represents the core of this paper, this topic will not be discussed further in this part of the contribution. We will deal with it in the next section.

### 3.6. Tracking

Target tracking provides a new estimation of the target location based on its foregoing positions. Target tracking usually results in the target trajectory estimation error decreasing, including trajectory smoothing. Most of tracking systems use a number of basic or advanced modifications of Kalman filters (e.g., linear, nonlinear and extended Kalman filters [47–49]) or particle filters [50,51]. Besides the fundamental theory of Kalman filtering and its modifications, further advanced methods of target tracking, such as single target and multiple target tracking systems [52,53] or multiple-hypothesis tracking methods [54], are available. They are based on the combination of data gating and data association and tracking, as well.

## 4. Cooperative Localization of the Target

In this section, we will deal with the problem of the cooperative localization of the target for the basic scenario. Firstly, the basic equations describing the target localization will be introduced. Then, TSM and JIEM as the possible approaches of the mentioned equation set solution will be derived.

### 4.1. Basic Equations

Let *TOA_R,i_* for *R* = *A*, *B*, *i* = 1, 2 represents the estimation of TOA of the electromagnetic wave transmitted by the *Tx_R_*, reflected by the target (*T* = (*x*, *y*)) and received by the *Rx_R,i_*. We presume that *TOA_R,i_* has been estimated by the algorithms outlined in the previous section. The distance, *d_R,i_*, between the transmitting antenna, *Tx_R_*, the target and the receiving antenna, *Rx_R,i_*, can then be expressed as:

(1) $$\begin{array}{cc} d_{R,i}=\text{cTO}A_{R,i} & R=A,B,i=1,2 \end{array}$$

where *c* is the propagation velocity of the electromagnetic wave emitted by the radar. In our consideration, *c* is set to the electromagnetic wave propagation velocity in air, *i.e.*, *c* = 3 ×10^8^ ms^−1^. On the other hand, the distance, *d_R,,i_*, can be expressed also by means of the antennas and target coordinates as follows:

(2) $$\begin{array}{ll} d_{R,i} & =r_{R,i}+e_{R,i}=\left\|Tx_{R},T\right\|+\left\|T,Rx_{R,i}\right\|+e_{R,i}\\ & =\sqrt{{\left(x-x_{R,t}\right)}^{2}+{\left(y-y_{R,t}\right)}^{2}}+\sqrt{{\left(x-x_{R,i}\right)}^{2}+{\left(y-y_{R,i}\right)}^{2}}+e_{R,i},\\ R & =A,B,i=1,2 \end{array}$$

In this expression, the symbol, ‖*XY*‖, is set for the Euclidean distance between the points, *X* and *Y*. The symbol:

(3) $$r_{R,i}=\left\|Tx_{R},T\right\|+\left\|T,Rx_{R,i}\right\|$$

expresses the true distance between the transmitting antenna, *Tx_R_*, the target and the receiving antenna, *Rx*_*R,i*_. Finally, *e*_*R,i*_ represents the additive noise component expressing the random errors of the TOA estimation.

Then, the target localization problem can be defined as the estimation of the target coordinates, (*x*, *y*), based on a solution of the set of four nonlinear Equations (2).

### 4.2. Taylor Series Method

The TSM method is a popular and very often used iterative method for object localization by UWB systems [19,55]. In TSM, a set of non-linear Equations (2) is linearized by expanding it in the Taylor series around a point corresponding to a guess of the solution of Equation (2) and only keeping terms below the second order. The obtained set of the linear equations is then solved by the least-squares method (LS) to produce an estimate of the target coordinates. The obtained solution is used in the next iteration as the new point of around which the Equation set (2) is linearized. The process then continues in a new iteration, until a predefined criterion is satisfied. Following this short outline, TSM for the solution of Equation (2) can be described as follows.

Let us define the functions:

(4) $$f_{R,i}(x,y)=\sqrt{{\left(x-x_{R,t}\right)}^{2}+{\left(y-y_{R,t}\right)}^{2}}+\sqrt{{\left(x-x_{R,i}\right)}^{2}+{\left(y-y_{R,i}\right)}^{2}}\;R=A,B,\;i=1,2$$

Then, the Equation set (2) can be rewritten as:

(5) $$f_{R,i}(x,y)=d_{R,i}-e_{R,i}\;R=A,B,\;i=1,2$$

where *e_R,i_* is the distance estimation error. If *x_v_* and *y_v_* are the initial guesses of the target location, then:

(6) $$\begin{array}{cc} x=x_{v}+\delta_{x}, & y=y_{v}+\delta_{y} \end{array}$$

In this expression, *δ_x_* and *δ_y_* are the location errors to be determined. Expanding *f*_*R,i*_(*x*, *y*) in the Taylor series and retaining the first two terms produces:

(7) $$f_{R,i}+a_{R,i,1}\delta_{x}+a_{R,i,2}\delta_{y}\approx d_{R,i}-e_{R,i}\;R=A,B,\;i=1,2$$

where:

(8) $$\begin{array}{ccc} f_{R,i}=f_{R,i}(x_{v},y_{v}), & a_{R,i,1}=\frac{\partial f_{R,i}(x,y)}{\partial x}{|}_{x=x_{v},y=y_{v}}, & a_{R,i,2}=\frac{\partial f_{R,i}(x,y)}{\partial y}{|}_{x=x_{v},y=y_{v}} \end{array}$$

By using Equation (8), the Equation (7) can be rewritten in matrix form as:

(9) Aδ=b−e

where:

(10) $$\begin{array}{cccc} \text{A}=\left[\begin{array}{cc} a_{A,1,1} & a_{A,1,2}\\ a_{A,2,1} & a_{A,2,2}\\ a_{B,1,1} & a_{B,1,2}\\ a_{B,2,1} & a_{B,2,2} \end{array}\right]\;, & \delta =\left[\begin{array}{c} \delta_{x}\\ \delta_{y} \end{array}\right]\;, & b=\left[\begin{array}{c} d_{A,1}-f_{A,1}\\ d_{A,2}-f_{A,2}\\ d_{B,1}-f_{B,1}\\ d_{B,2}-f_{B,1} \end{array}\right]\;, & e=\left[\begin{array}{c} e_{A,1}\\ e_{A,2}\\ e_{B,1}\\ e_{B,2} \end{array}\right] \end{array}$$

Then, the LS estimation of *δ* is given by:

(11) $$\delta ={\left({\text{A}}^{\text{T}}\text{A}\right)}^{-1}{\text{A}}^{\text{T}}\text{b}$$

and the target coordinates are updated in the iteration process according to:

(12) $$\begin{array}{cc} x_{v}\leftarrow x_{v}+\delta_{x}, & y_{v}\leftarrow y_{v}+\delta_{y} \end{array}$$

The updated estimation of the target coordinates according to (Equation (12)) is used as the new guess of the target coordinates for the next iteration. The iteration process (Equations (8)–(12)) is repeated until the condition:

(13) $$\left\|\delta^{\left(i\right)}\right\|<\epsilon$$

is satisfied. In this expression, ‖*δ*^(i)^‖ and *ϵ* are the Euclidean norm of the location error vector at the *i^th^* iteration and a positive small constant, sometimes referred to as the termination parameter, respectively. On the other hand, if:

(14) $$\left\|\delta^{\left(i\right)}\right\|>\left\|\delta^{\left(i+1\right)}\right\|$$

is true, the iteration process is not converging. In this case, a new initial guess has to be set up, or the result of the *i^th^* iteration step is taken as the target coordinate estimation.

### 4.3. Method of Joining Intersections of the Ellipses

Let us assume for a moment a perfect estimate of *TOA*_*R,i*_, i.e., if *e*_*R,i*_ = 0. Then, the Equation set (2) will take the following form:

(15) $$d_{R,i}=cTOA_{R,i}=\left\|Tx_{R},T\right\|+\left\|T,Rx_{R,i}\right\|\;R=A,B,\;i=1,2$$

This expression represents the equation of the ellipse with the foci, *Tx_R_*= (*x*_*R,t*_, *y*_*R,t*_) and *Rx_R,i_* = (*x_R,i_*, *y_R,i_*), and with the length of the semi-major axis, *d_R,i_*/2. Thus, there is a family of the ellipses for each *Tx_R_*-*Rx_R,1_* pair (*R* = *A*, *B*, *i* = 1, 2) with the foci in *Tx_R_* and *Rx_R,i_*, for all possible values of *d_R,i_* [56]. Since the target coordinates have to satisfy Equation (15) and the coordinates of the transmitting and receiving antennas are known, the target coordinates can be determined as the intersection of the ellipses formed by two different *Tx_R_* — *Rx_R,i_* pairs. These intersections can be obtained as the solution of a couple of the corresponding nonlinear Equations of (15). If the target coordinates are computed as the intersection of two ellipses, then this approach is referred to as DC [7,9]. Generally, the number of the intersections of two ellipses can take on values of zero, one, two, three or four. However, not each intersection expresses the target position. Therefore, the intersection corresponding to the target has to be carefully selected. The detailed description of the analytical calculation of the intersections of two ellipses is not provided in this paper. The comprehensive solution of this mathematical task can be found, e.g., in [57].

**Figure 2.**
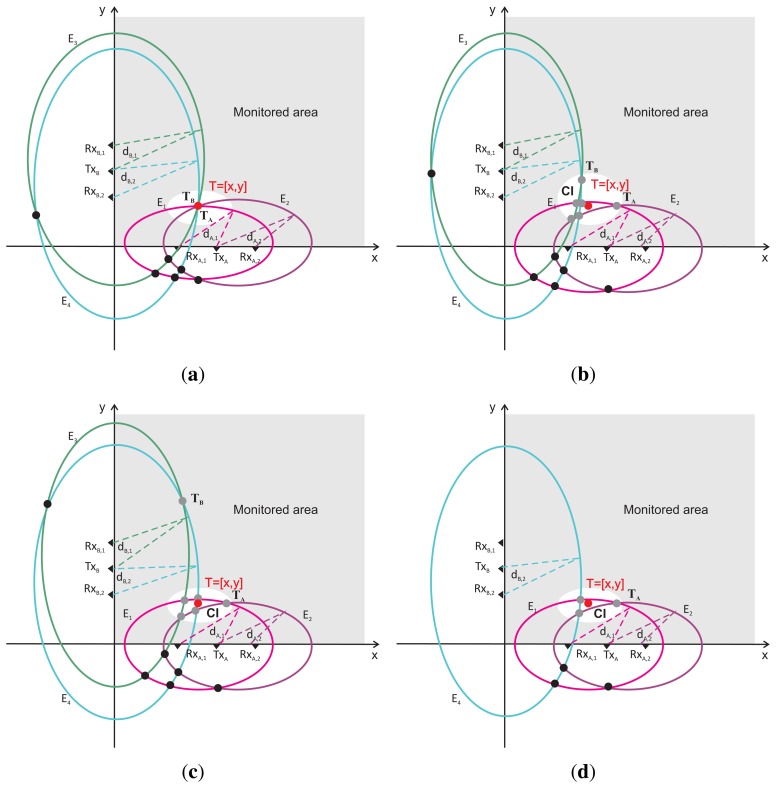
Geometrical interpretation of target localization. Review of basic scenarios. (**a**) Perfect estimations of time-of arrival (TOA) by *RS_A_* and *RS_B_*; (**b**) good estimations of TOA by *RS_A_* and *RS_B_*; (**c**) good estimations of TOA by *RS_A_*; pure estimations of TOA by *RS_B_*; (**d**) good estimations of TOA by *RS_A_*; *RS_B_* is not able to localize a target.

Let us return now to the basic scenario outlined in Figure 1. By using *TOA_R,i_* for *R* = *A*, *B*, *i* = 1, 2, four ellipses, *E_i_*, for *i* = 1, 2, 3, 4, can be constructed. Their foci $F_{1}^{\left(i\right)}$, $F_{2}^{\left(i\right)}$, and the lengths of their semi-major axis, *a_i_*, are given in Table 1. A possible system of the ellipses, *E_i_*, for the perfect estimates of *TOA_R,i_* is sketched in Figure 2a. In this figure, it can be seen that there is only one joint intersection of all ellipses. This intersection represents the solution of Equation (2) and, at the same time, the target position.

Unfortunately, *TOA_R,i_* is normally never estimated with the zero error. The scenario for not perfect, but good, estimations of *TOA_R,i_* for *R* = *A*, *B*, *i* = 1, 2 is outlined in Figure 2b. The target position estimated by RS_A_ labeled as *T_A_*, is given by the intersection of *E*_1_ and *E*_2_. On the other hand, the intersection of *E*_3_ and *E*_4_, labeled as *T_B_*, is set for the target position estimated by RS_B_. It can be observed from Figure 2b that *T_A_* and *T_B_* are quite close to the true target position, *T*. However, in the case of through-the-wall target localization, the heavy clutter and shadowing effect, due to the wall and static objects located within the monitored area, are present. This results in that TOA is very frequently estimated with large error or eventually the target is not detected. The former case is sketched in Figure 2c, where the target position estimated by RS_B_ (*T_B_*) is very poor. The latter scenario is outlined in Figure 2d. Since the target was not detected by *Rx_B_*_,1_, it was not possible to create the ellipse, *E*_3_, and hence, RS_B_ was not able to localize the target. In both scenarios, better results can be provided by RS_A_ (Figure 2c,d).

The analyses of the scenarios given in Figure 2b-d indicate that a single radar system may not be able to provide very accurate and robust estimation of the target position. It is true especially for through-the-wall target localization, which has been confirmed by quite a number of measurements. On the other hand, it can be identified from Figure 2b-d that there are still several additional intersections of the ellipses (not only *T_A_* and *T_B_*), which are localized near the true position of the target, and hence, they have a potential to be used for target positioning. The set of such intersections can be jointed to create the cluster of the intersections (*CI*) proper for the target localization. The main idea of JIEM presented in this paper is to create such *CI* and, subsequently, to use the intersections joint in *CI* for target positioning. For *CI* creation, the novel decision algorithm will be used. Following this idea, JIEM can be described as follows.

Generally, four ellipses, *E_i_*, for *i* = 1, 2, 3, 4, represented by Equation (2), will be created at the start of JIEM. Let us define set *CI* of the points in the monitored area with the initial setting, *CI* = ∅. Let us create the pairs of the ellipses, (*E*_1_, *E*_2_), (*E*_1_, *E*_3_), (*E*_1_, *E*_4_), (*E*_2_, *E*_3_), (*E*_2_, *E*_4_) and (*E*_3_, *E*_4_). For every pair of the ellipses, *P_ij_* = (*E_i_*, *E_j_*), the ellipse intersections will be computed according to the algorithm described in [57]. Let us denote all intersections of the ellipses, *E_i_*, *E_j_*, as $T_{k}^{\left(i,j\right)}=\left(x_{k}^{\left(i,j\right)},y_{k}^{\left(i,j\right)}\right)$ for *k* = 1, 2, …, *max_k_*. Variable *max_k_* ∈ {0, 1, 2, 3, 4} denotes the ellipse intersection number. Hereinafter, only those intersections located in the monitored area will be taken into account. If the ellipses have no intersection, a new pair of the ellipses will be taken for *CI* creation. If the ellipses, *E_i_* and *E_j_*, are touching, then touching point $T_{1}^{\left(i,j\right)}$ will be put into set *CI*. If the ellipses, *E_i_*, *E_j_*, have more intersections, the decision algorithm will be used for the selection of their intersections situated closest to other ellipses. This intersection will be included in *CI*.

The decision algorithm can be described as follows. Let us consider the ellipses, *E_m_* and *E_n_*, for *m* < *n*. Now, the task is to determine the intersection of *E_l_* and *E_k_* for *l* ≠ *m*, *n* and *k* ≠ *m*, *n* (*i.e.*, the intersection not found on *E_m_* and *E_n_*) situated closest to *E_m_* and *E_n_*. For that purpose, two points, *Q_m_* and *Q_n_*, from the intersections, $T_{k}^{\left(i,j\right)}$, are selected according to:

(16) $$Q_{m}=\left\{T_{k}^{\left(i,j\right)}:\underset{k=1,\ldots ,\max_{k}}{\arg\min}\left(\left|\left\|F_{1}^{\left(m\right)},T_{k}^{\left(i,j\right)}\right\|+\left\|T_{k}^{\left(i,j\right)},F_{2}^{\left(m\right)}\right\|-2a_{m}\right|\right)\right\}\;i,j\in \left\{1,2,3,4\right\},\;i\neq m,i\neq j$$

(17) $$Q_{n}=\left\{T_{k}^{\left(i,j\right)}:\underset{k=1,\ldots ,\max_{k}}{\arg\min}\left(\left|\left\|F_{1}^{\left(n\right)},T_{k}^{\left(i,j\right)}\right\|+\left\|T_{k}^{\left(i,j\right)},F_{2}^{\left(n\right)}\right\|-2a_{n}\right|\right)\right\}\;n\neq m,i,j\in \left\{1,2,3,4\right\},\;i\neq m,i\neq j$$

where *a_v_* are given in Table 1.

The main idea of Equations (16) and (17) consists in the application of the metrics:

(18) $$M\left(E_{v},T_{k}^{\left(i,j\right)}\right)=\left|\left\|F_{1}^{\left(v\right)},T_{k}^{\left(i,j\right)}\right\|+\left\|T_{k}^{\left(i,j\right)},F_{2}^{\left(v\right)}\right\|-2a_{v}\right|$$

expressing a measure of the closeness of $T_{k}^{\left(i,j\right)}$ to *E_v_*. It follows from Equation (18) that if $T_{k}^{\left(i,j\right)}$ is situated on *E_v_*, then $M\left(E_{v},T_{k}^{\left(i,j\right)}\right)=0$. If $T_{k}^{\left(i,j\right)}$ is not situated on *E_v_*, then an ellipse crossing $T_{k}^{\left(i,j\right)}$ having the same foci as *E_v_* and a semi-major axis, *ā_v_*, can be created. Then, the less |*a_v_* − *ā_v_|* is, the closer $T_{k}^{\left(i,j\right)}$ to *E_v_* is situated.

Taking into account the significance of the metrics Equation (18), we can see that *Q_m_* is the intersection situated closest to *E_m_*, but not located on this ellipse. On the other hand, *Q_n_* is the intersection situated closest to *E_n_*, but not located on *E_m_* and *E_n_*. Hence, if *Q_m_* = *Q_n_*, *Q_m_* is at once closest to *E_m_* and *E_n_*. Therefore, if *Q_m_* = *Q_n_*, then the point, *Q_m_*, will be put in *CI*. On the other hand, if *Q_m_* ≠ *Q_n_*, *Q_m_* and *Q_n_* will not be included in *CI*.

The described decision algorithm will be applied step by step for all pairs of the ellipses, *P_mn_*, for *m* < *n*. As a result, the complete *CI* will be obtained. Then, the target coordinates are estimated as the arithmetical average of the corresponding coordinates of the points of *CI*. As the introduced JIEM exploits TOA estimated by both radar systems, it belongs to the cooperative methods of target localization.

For the basic scenario, there are also situations when the four ellipses cannot be created. Then, the following approach is used by JIEM. If there are only three ellipses with more than one intersection in the monitored area, the target position is estimated as the intersection of the ellipses belonging to the same radar system. If there is only one intersection in the monitored area, its coordinates represent the estimated coordinates of the target. Finally, if at least two ellipses cannot be created or if no intersection can be found in the monitored area, the target cannot be localized.

## 5. Experimental Results

**Figure 3.**
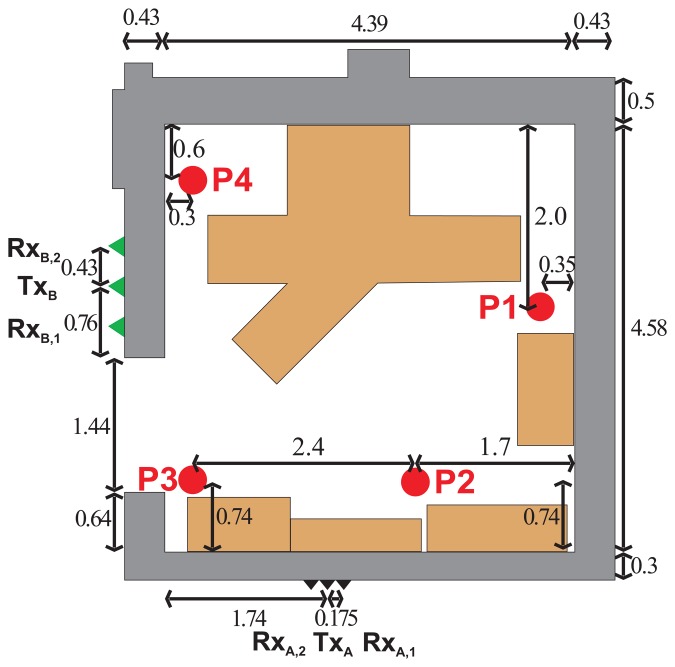
The scheme of measurement for the basic scenario.

**Figure 4.**
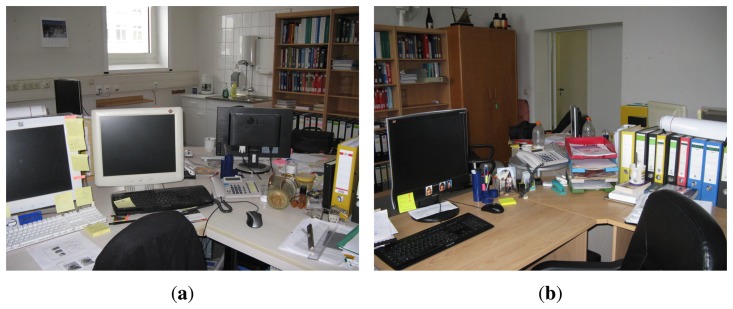
Room interior. (**a**) View from reference position P4; (**b**) View from behind reference position P1.

In order to evaluate JIEM performance, the scenario outlined in Figure 3 is analyzed. The scenario is represented by a tracking of a person moving behind the brick walls using two independent M-sequence UWB radar systems (Figure 4). The person was moving with approximately constant speed. The thickness of the first and the second wall was 30 cm and 43 cm, respectively. The person to be localized and tracked was walking inside a fully furnished room (Figure 4) from position P1 through positions P2 and P3, up to position P4 (Figure 3).

**Figure 5.**
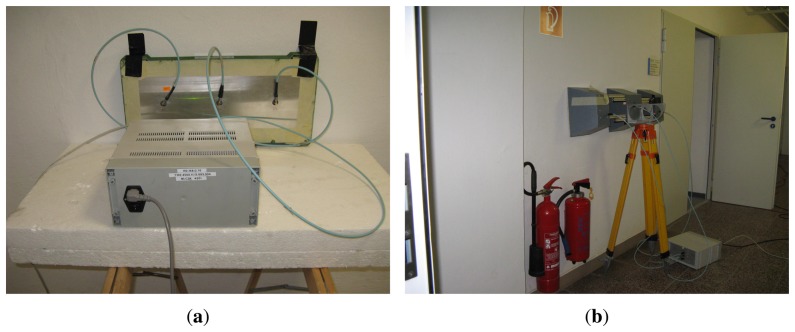
M-sequence UWB radar systems with (**a**) spiral antennas; (**b**) horn antennas.

The raw radar data analyzed in this contribution were acquired by means of two M-sequence UWB radar systems (Figure 5), each equipped with one transmitting and two receiving antennas [4,5]. The radar antenna positions are outlined in Figure 3. The system clock frequency of both radar devices are about 4.5 GHz, which results in the operational bandwidth of about DC-2.25 GHz. The order of the M-sequence emitted by the radar is nine, *i.e.*, the impulse response covers 511 samples regularly spread over 114 ns. This corresponds to an observation window of 114 ns, leading to an unambiguous range of about 17 m. Two-hundred fifty-six hardware averages of the environment impulse responses are always computed within the radar head field-programmable gate array (FPGA) to provide a reasonable data throughput and to improve the signal-to-noise ratio (SNR) by 24 dB. The additional software averaging can be provided by the basic software of the radar device. In our measurement, the radar systems were set in such a way as to provide approximately 10 impulse responses per second, *i.e.*, the radar devices have generated the impulse responses with the period of *T_RD_* = 100 ms. The total power transmitted by the particular radars was about 1 mW.

The first radar system, denoted as RS_A_, has been equipped with three spiral antennas (Figure 5a) placed on the wall (Figure 3). The second radar system, denoted as RS_B_, has been equipped with three double-ridged horn antennas (Figure 5b) placed on the wall, too (Figure 3). Here, transmitting antennas have been located in the middle between two receiving antennas. The distances between adjacent antennas were set to 17.5 cm and 43 cm for RS_A_ and RS_B_, respectively.

In the case of our measurements, the synchronization of RS_A_ and RS_B_ has been created by the interconnection of RS_A_ and RS_B_ through the local area network (LAN). The outlined application of LAN has allowed one to start the measurement of the particular impulse responses by RS_A_ and RS_B_ theoretically in the same time instant (practically, almost in the same time). No additional signal processing has been applied for the purpose of the synchronization of the data provided by RS_A_ and RS_B_. Taking into account the rate of measurement (10 impulse response per second) and the speed of the target movement (approximately 0.80–1.00 ms^−1^), we believe that this form of RS_A_ and RS_B_ synchronization can be acceptable for the cooperative localization of the moving target.

In the basic scenario, both radar systems have operated simultaneously over the same frequency band. They have emitted the same M-sequence, but the initial conditions of their M-sequence generators have been set randomly. Hence, the M-sequences generated by the first and second radar system are theoretically the same, but they are mutually shifted. Because the radar receiver is based on an application of the correlation between the transmitting M-sequence and the received signal, we have expected that it should result in additional correlation peaks, due to the transmitting of the second radar system. These peaks should be delayed only according to the initial conditions in comparison to the first radar system. However, this effect and, therefore, no mutual interference of the radar systems have been identified in our measurement. The deeper analyses of this effect provided by [58] have shown that the mentioned effect has its origin in the difference between the system clock frequency of the particular radars. It has been demonstrated in [58] that if the difference between the system clock frequency of both radars is greater than several kHz only, the correlation between two M-sequences generated by these radar systems is canceled, and hence, the possible mutual interference of both radar systems is negligible. This property of the M-sequence UWB radar systems has been exploited to an advantage within our measurement.

The raw radar data acquired by the described measurement have been processed by the procedure described in Section 3. Here, the exponential averaging method, CFAR detector, the method introduced in [26] and linear Kalman filters (KF) have been applied for the background subtraction, target detection, TOA estimation and target tracking, respectively. For the target localization by the single RS_A_ and RS_B_, the DC method has been used. On the other hand, MEAN, TSM and JIEM have been applied for cooperative positioning of the moving target by using two independent radar systems.

It is well known that TSM performance depends on the initial guess of the target location (*T*(*x_v_*, *y_v_*)) for the particular iterations and on the number of iterations [12,18]. In the case of the analyzed scenario, the least-mean square method [24], the weighted arithmetic average of the target positions estimated by RS_A_ and RS_B_ [24] and JIEM were tested for the estimation of the mentioned initial guess. Here, the best performance was provided by JIEM. Therefore, all results presented in this contribution for TSM were obtained for the initial estimate of the target location by JIEM. The number of iterations was controlled by the condition (13). The real number of iterations was not greater than 10.

**Figure 6.**
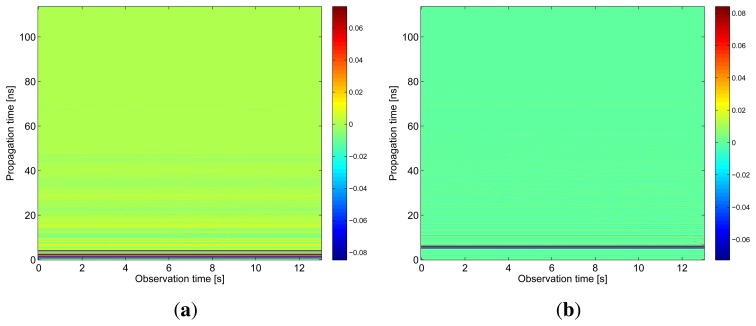
Radargram with preprocessed raw radar signals. (**a**) The first receiving channel of RS_A_; (b) the first receiving channel of RS_B_.

**Figure 7.**
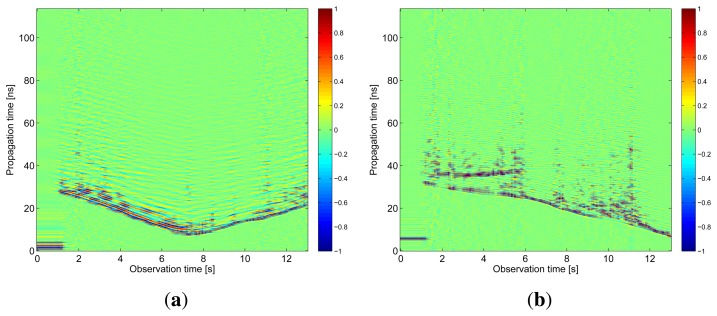
Radargram with the subtracted background. (a) The first receiving channel of *RS_A_*; (**b**) the first receiving channel of *RS_B_*.

**Figure 8.**
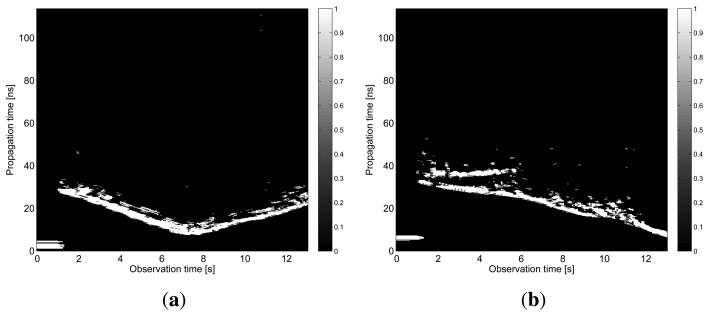
Detector output. (**a**) The first receiving channel of *RS_A_*; (**b**) the first receiving channel of *RS_B_*.

**Figure 9.**
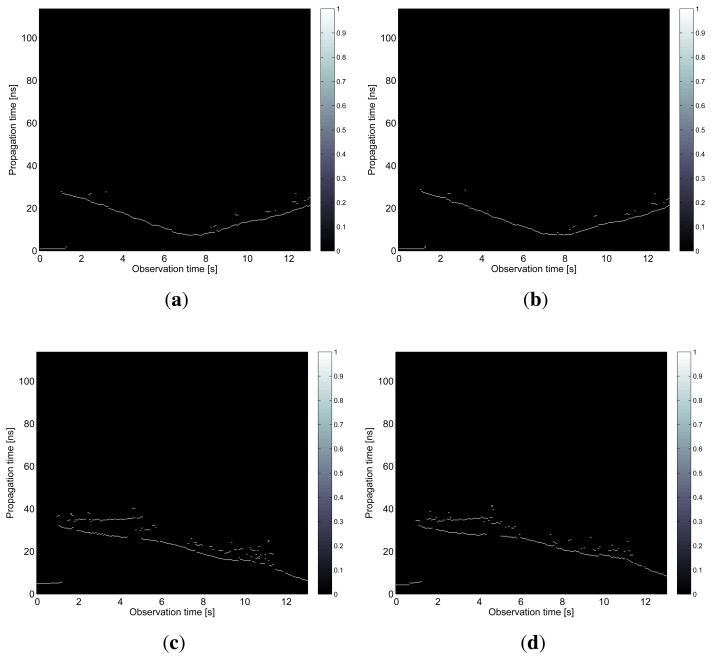
TOA estimations. (**a**) The first receiving channel of RS_A_; (**b**) the second receiving channel of *RS_A_*; (**c**) the first receiving channel of *RS_B_*; (**d**) the second receiving channel of *RS_B_*.

**Figure 10.**
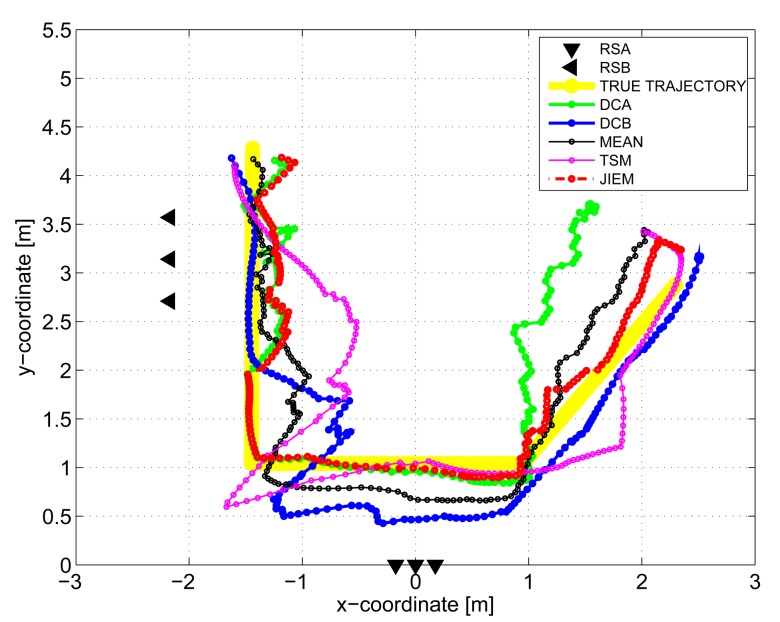
Target tracks estimated by all considered methods.

**Figure 11.**
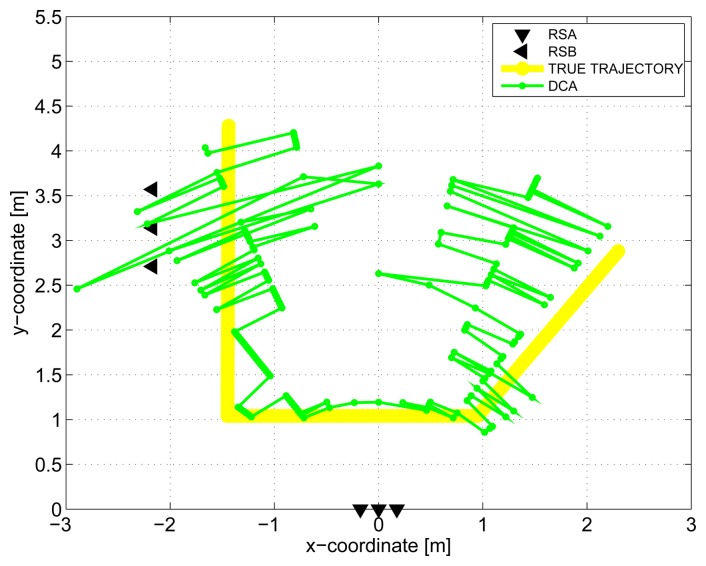
Target trajectory estimated by DCA.

**Figure 12.**
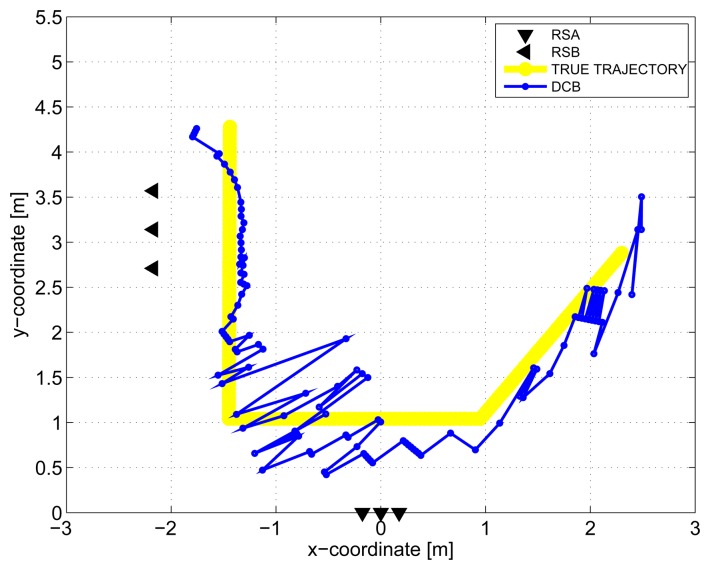
Target trajectory estimated by DCB.

**Figure 13.**
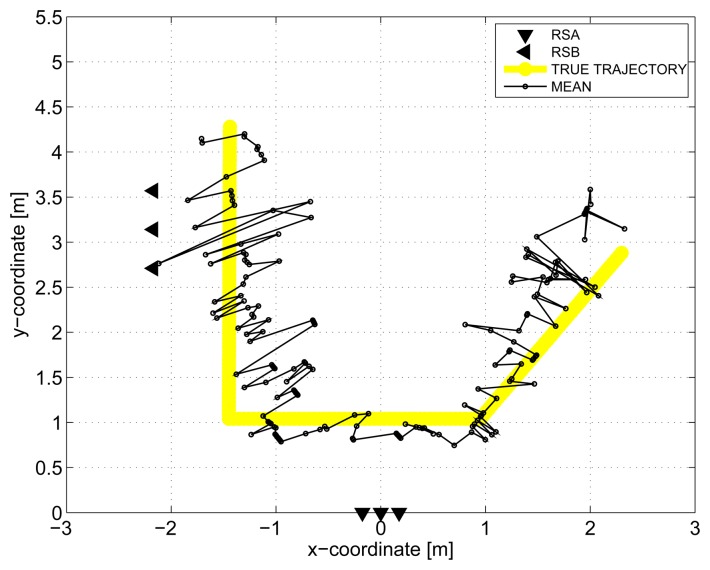
Target trajectory estimated by MEAN.

**Figure 14.**
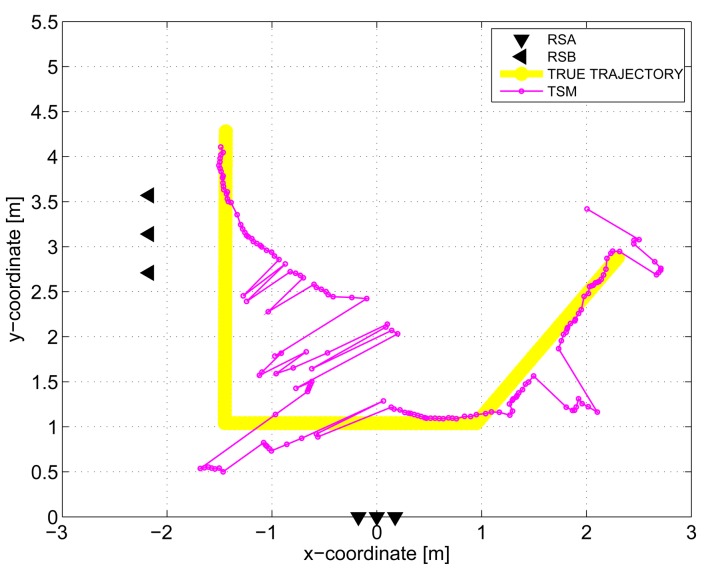
Target trajectory estimated by Taylor-Series method (TSM).

**Figure 15.**
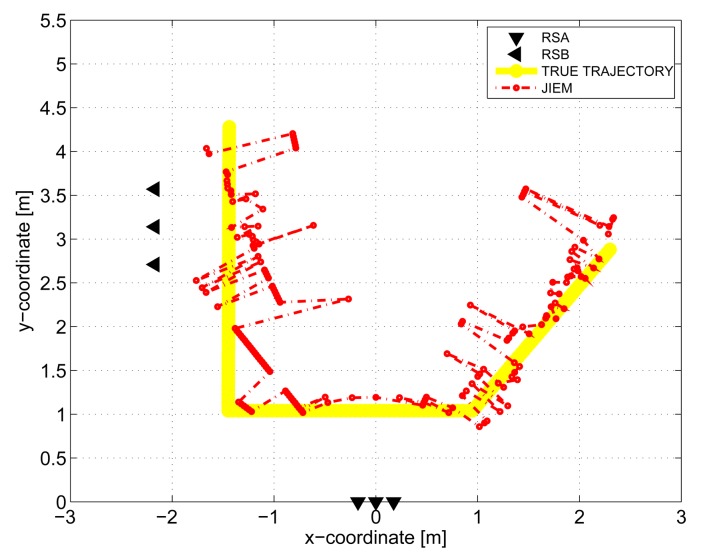
Target trajectory estimated by joining intersections of the ellipses (JIEM).

The results obtained in the particular phases of UWB radar signal processing for the basic scenario are given in Figures 6, 7, 8, 9 and 10. The raw radar data, the radargrams with the subtracted background and the detector outputs for the first receiving channels of RS_A_ and RS_B_ are given in Figures 6, 7 and 8. TOA estimations for all receiving channels are depicted in Figure 9. The target trajectory estimations as localization phase output are given in Figures 11, 12, 13, 14 and 15. Here, the trajectories estimated by the direct calculation method applied on data from RS_A_ and RS_B_ are denoted as DCA and DCB, respectively. Finally, the target tracks obtained by KF applied to the target trajectories obtained by DC (KF DCA, KF DCB), MEAN (KF MEAN), TSM (KF TSM) and JIEM (KF JIEM) are given in Figure 10.

**Figure 16.**
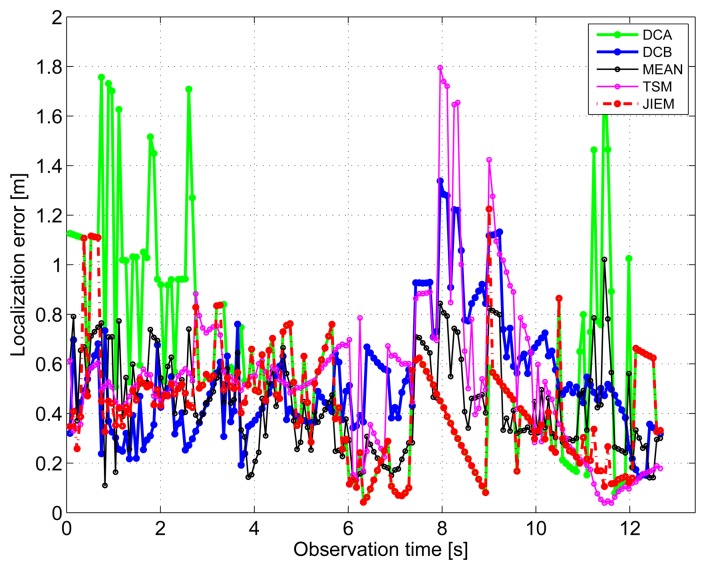
Localization errors for all estimated trajectories.

**Figure 17.**
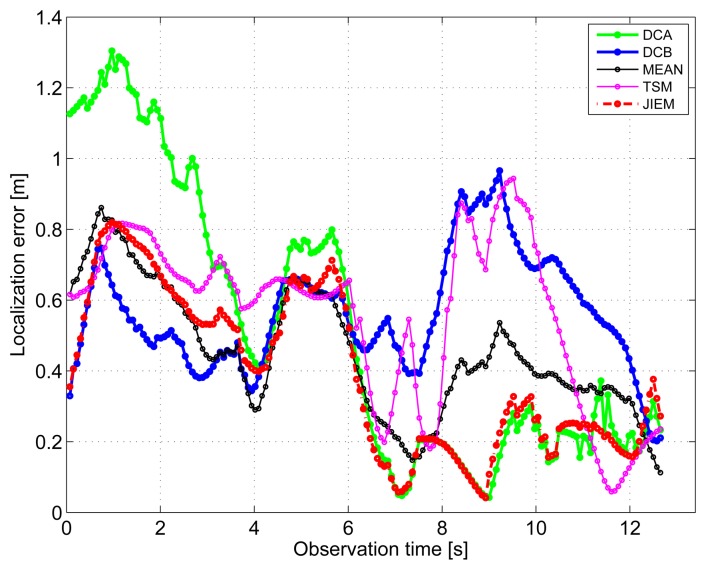
Localization errors for all estimated tracks.

The target position estimation accuracy corresponding to the particular methods of target localization is illustrated also by the time evolution of target localization errors for the estimated trajectories and tracks (Figures 16 and 17). The mean and the root mean square (RMS) values of the target localization errors for the estimated trajectories and tracks are given in Tables 2 and 3.

Now, after this short summary of the obtained results, we can discuss some outcomes in detail. Let us begin with the target trajectories received as the localization phase output by DC for the RS_A_ and RS_B_ (Figures 11 and 12). These figures show that the estimated trajectories follow the target motion direction, but with a high deviation. As we mentioned in Section 4, the target localization by the DC method consists of the solution of the set of two nonlinear equations. If the solution exists, the target is localized at the intersections of two ellipses. It follows from this geometrical interpretation of the target positioning that its accuracy depends strongly on the angle between the tangent lines of both ellipses, where the tangent point is their intersection. For the angle of around 90°, the intersection of the ellipses can be computed with high accuracy. On the other hand, for the angle of around 0° (*i.e.*, the ellipses are almost touching), the ellipse intersection coordinates cannot be computed accurately, due to the numerical error of the computation. The closer the transmitting and receiving antennas are, the greater this effect is. In the case of the target localization by the DC method, the sizes of the semi-major axes of the ellipses are given by the TOA estimations. Then, the accuracy of the target positioning is very sensitive to the precision of TOA estimation, especially if the mentioned angle between the tangent lines of both ellipses is small. This effect manifests itself in such a way that if the mentioned angle is small, the small errors of TOA estimation will result in a large error of the target coordinate estimation. The combination of this effect with the impact of the monitored area quantization at the M-sequence radar application [59] will result in that the target trajectory estimation by DC will be obtained, with the error component having a high variance.

The improved accuracy of the target localization can be obtained by MEAN, *i.e.*, by a simple combination of the target coordinates provided by DCA and DCB. The improved performance of MEAN illustrated in Figure 13 is especially due to the mutual position of RSA and RSB and the setting of the true track of the target.

The target trajectory estimated by TSM within the localization phase is presented in Figure 14. Here, the pure performance of TSM can be observed. This can be explained by the fact that TSM is based on the solution of the Equation set (2). Since the number of the Equations is small, the solution is very sensitive to TOA estimation. Therefore, if leastwise, only one TOA is estimated with a large error, the target position will be also estimated with a large error. Unfortunately, in the case of through-the-wall target positioning, TOA estimation can be obtained many times with a large error, due to heavy clutter and a complex environment. This results in the pure performance of TSM in spite of the fact that the outputs of both radar systems are used for cooperative positioning of the target. On the other hand, we expect that TSM performance could be improved if more radar systems are used for the target localization.

The comparison of the target trajectory estimations by the localization methods considered in this paper has shown that the best performance is provided by JIEM (Figure 15).

The standard approach for how to improve the target trajectory estimation obtained by the localization phase is to apply tracking filters [53]. The final tracks corresponding to DCA, DCB, MEAN, TSM and JIEM application obtained by target tracking using KF are depicted in Figure 10 as the curves, KF DCA, KF DCB, KF MEAN, KF TSM and KF JIEM, respectively. Here, the presented tracks have shown the significant improvement of the target trajectory estimation in comparison with that given in Figures 11, 12, 13, 14 and 15. If we compare the estimates of the target trajectories and tracks given in Figures 11–10, we can conclude that the best estimate of the target trajectory and track is provided by JIEM. This conclusion is also confirmed by the time evolution of target localization errors for the estimated trajectories and tracks (Figures 16 and 17) and by the mean and RMS values of the time evolution of localization errors (Tables 2 and 3). In the case of the time evolution computation of the estimation errors, the presumption of the constant speed of the target movement has been applied. The mentioned JIEM performance follows from the fact that JIEM exploits only the carefully selected intersections of the particular ellipses (*i.e.*, *CI*) for the target coordinate evaluation.

The performance properties of JIEM have been tested also in [25]. The analyzed scenario has been represented by through-the-wall localization and tracking of a person moving in a classroom. The performance properties of JIEM have been evaluated by the visual inspection of the target tracks (the shape and the localization of the true and estimated target tracks) and by the alternative form of the target position estimation accuracy evaluation, referred to as “the percentage of the true estimates of the target positions”. The results presented in [25] have shown and confirmed that JIEM has provided better performance than the other tested methods, also for a scenario different from that described in this paper.

The described procedure of signal processing has been implemented on a standard notebook using MATLAB software. In Table 4, the so-called average time of the calculation (*T_L_*) is brought out. This quantity represents the average time of the calculation of the target coordinates by using DC, MEAN, TSM and JIEM of the estimated TOA are their inputs. Therefore, *T_L_* can be taken as an approximate measure of the described algorithm complexity. The average time needed for radar signal processing within the background subtraction, target detection, TOA estimation and the tracking phase is *T*_0_ = 12 ms. Then, the total average time necessary for the target position evaluation corresponding to one impulse response processing is *T_SP_* = *T_L_* + *T*_0_. As follows from Table 4, the best performance of JIEM in comparison with that of DC, MEAN and TSM is reached at the cost of its high complexity. In the case of JIEM, the time intervals, *T_L_* and *T_SP_*, are 4.85 ms and 16.85 ms, respectively. As the radar devices have generated the impulse responses with the period of *T_RD_* = 100 ms, we can conclude that a relatively high value of *T_L_* for JIEM can be accepted.

## 6. Conclusions

This paper has been devoted to target localization by two independent UWB radar systems. A novel method of the cooperative localization of the target, referred to as JIEM, has been proposed. The main idea of JIEM consists in the creation of *CI*, which is applied to the target coordinate estimation. The performance properties of JIEM have been compared with DC, MEAN and TSM. For that purpose, the scenario of through-the-wall tracking of a moving target has been analyzed. The obtained results have shown that JIEM has overcome other considered methods of target localization at the cost of its high, but still acceptable, complexity.

JIEM can be used for cooperative target positioning with an advantage, especially if the number of radar systems applied for target positioning is not very high. This application scenario represents a trade-off between its good performance and high complexity. On the other hand, conventional methods, such as DC, MEAN or TSM, have a limited ability to provide a robust performance for through-the-wall localization of moving persons. Their high sensitivity to TOA estimation errors is the main reason for their pure performance in such scenarios. Taking into account these facts, JIEM should be preferred for through-the-wall target localization by two radar systems in comparison with that of DC, MEAN or TSM.

JIEM possess the potential to also be easily extended for target localization by a UWB sensor network consisting of more than two sensors, for target localization by a UWB multistatic radar system with *N* > 2 receiving antennas or for person positioning based on detection of his or her breathing by UWB radar systems [60–62]. In these scenarios, a set of TOAs corresponding to each pair of transmitting and receiving antennas can be estimated, and an adequate set of the ellipse pairs can be created. Then, JIEM can be directly applied.

We assume that further improvement of JIEM performance can be obtained by its non-complex modifications. The promising modification of JIEM can be seen in the target coordinates estimation based on the intersections included in *CI*. Here, the simple arithmetic average used in the current version of JIEM can be replaced by a more powerful algorithm (e.g., least-square method). Another useful modification of JIEM can be created, e.g., by its adaptation to the scenario in which only three ellipses can be created (Figure 2d). The suggested extensions and modifications of JIEM can be considered to be a promising topic for our follow up research in the field of through-the-wall person localization by UWB sensor networks.

## Figures and Tables

**Table 1.** Parameters of ellipses.

**Ellipse *E****_i_*	**Focus $F_{1}^{\left(i\right)}$**	**Focus $F_{2}^{\left(i\right)}$**	**Semi-Major Axis *a****_i_*
*i* = 1	*Rx_A_*_,1_ = (*x_A_*_,1_,*y_A_*_,1_)	*Tx_A_* = (*x_A,t_*, *y_A,t_*)	*d_A_*_,1_/2
*i* = 2	*Rx_A_*_,2_ = (*x_A_*_,2_,*y_A_*,_2_)	*Tx_A_* = (*x_A,t_*, *y_A,t_*)	*d_A_*_,2_/2
*i* = 3	*Rx_B_*_,1_ = (*x_B_*_,1_,*y_B_*_,1_)	*Tx_B_* = (*x_B,t_*, *y_B,t_*)	*d_B_*_,1_/2
*i* = 4	*Rx_B_*_,2_ = (*x_B_*_,2_,*y_B_*_,2_)	*Tx_B_* = (*x_B,t_*, *y_B,t_*)	*d_B_*_,2_/2

**Table 2.** Mean and RMS values of target localization errors for the estimated trajectories.

**Trajectories**	**DCA**	**DCB**	**MEAN**	**TSM**	**JIEM**
Mean [m]	0.5846	0.5342	0.4418	0.5445	0.4215
RMS [m]	0.7025	0.5858	0.4818	0.6304	0.4776

**Table 3.** Mean and RMS values of target localization errors for the estimated tracks.

**Tracks**	**DCA**	**DCB**	**MEAN**	**TSM**	**JIEM**
Mean [m]	0.5187	0.5644	0.4546	0.5606	0.3961
RMS [m]	0.6470	0.5865	0.4881	0.6065	0.4558

**Table 4.** Illustration of computational complexity of target localization by DC, MEAN, TSM and JIEM.

**Localization Methods**	**DCA**	**DCB**	**MEAN**	**TSM**	**JIEM**
Average time of calculation [ms]	0.18	0.18	0.37	5.79	4.85

## References

[1] Withington P., Fluhler H., Nag S. (2003). Enhancing homeland security with advanced UWB sensors. IEEE Microw. Mag..

[2] Amin M., Sarabandi K. (2009). Special issue on remote sensing of building interior. IEEE Trans. Geosci. Remote Sens..

[3] Fishler E., Haimovich A., Blum R., Cimini L., Chizhik D., Valenzuela R. (2006). Spatial diversity in radars-models and detection performance. IEEE Trans. Signal Process..

[4] Sachs J., Aftanas M., Crabbe S., Drutarovský M., Klukas R., Kocur D., Nguyen T., Peyerl P., Rovňáková J., Zaikov E. Detection and Tracking of Moving or Trapped People Hidden by Obstacles Using Ultra-Wideband Pseudo-Noise Radar.

[5] Daniels D. (2004). M-Sequence Radar. Ground Penetrating Radar.

[6] Rovňaková J. (2010). Complete Signal Processing for Through Wall Tracking of Moving Targets.

[7] Kocur D., Rovňáková J., Švecová M., Rudas I.J., Fodor J., Kacprzyk J. (2009). Through Wall Tracking of Moving Targets by M-Sequence UWB Radar. Towards Intelligent Engineering and Information Technology.

[8] Rovňáková J., Švecová M., Kocur D., Nguyen T.T., Sachs J. Signal Processing for Through Wall Moving Target Tracking by M-Sequence UWB Radar.

[9] Švecová M., Kocur D., Zetik R. Object Localization Using Round Trip Propagation Time Measurements.

[10] Švecová M. Node Localization Methods in UWB Wireless Sensor Networks: A Review.

[11] Chong E.K.P., Żak S.H. (2008). An Introduction to Optimization.

[12] Yu K., Saarnisaari H., Montillet J.-P., Rabbachin A., Oppermann I., de Abreu G.T.F. (2006). Ultra-Wideband Wireless Communications and Networks.

[13] Sayed A.H., Tarighat A., Khajehnouri N. (2005). Network-based wireless location: Challenges faced in developing techniques for accurate wireless location information. IEEE Signal Process. Mag..

[14] Cheung K., So H., Ma W.K., Chan Y.T. (2004). Least squares algorithms for time-of-arrival-based mobile location. IEEE Trans. Signal Process..

[15] Huang Y., Benesty J., Elko G.W., Mersereati R.M. (2001). Real-time passive source localization: A practical linear-correction least-squares approach. IEEE Trans. Speech Audio Process..

[16] Smith J.O., Abel J.S. (1987). The spherical interpolation method of source localization. IEEE J. Ocean. Eng..

[17] Smith J.O., Abel J.S. (1987). Closed-form least-squares source location estimation from range-difference measurements. IEEE Trans. Acoust. Speech Signal Process..

[18] Foy W.H. (1976). Position-location solutions by Taylor-series estimation. IEEE Trans. Aerosp. Electron. Syst..

[19] Oppermann I., Hamalainen M., Iinatti J. (2004). UWB Theory and Applications.

[20] Bartoletti S., Giorgetti A., Conti A. UWB Sensor Radar Networks for Indoor Passive Navigation.

[21] Chiani M., Giorgetti A., Mazzotti M., Minutolo R., Paolini E. Target Detection Metrics and Tracking for UWB Radar Sensor Networks.

[22] Shen J., Molisch A.F., Salmi J. (2012). Accurate passive location estimation using toa measurements. IEEE Trans. Wirel. Commun..

[23] Giorgetti A. (2013). Time-of-arrival estimation based on information theoretic criteria. IEEE Trans. Signal Process..

[24] Švecová M. (2009). Target Localization by UWB Radar System. Ph.D. Thesis.

[25] Švecová M., Kocur D. Target Localization by the Method of Joining Intersections of the Ellipses.

[26] Rovňáková J. (2009). Complete Signal Processing for Through Wall Target Tracking by M-Sequence UWB Radar System. Ph.D. Thesis.

[27] Kocur D., Rovňáková J., Geogiadis A., Rogier H., Roselli L., Arcioni P. (2012). Short-Range Tracking of Moving Targets by Handheld UWB Radar System. Microwave and Milimeter Wave Circuits and Systems—Emerging Design, Technologies and Applications.

[28] Piccardi M. Background Subtraction Techniques: A Review.

[29] Zetik R., Crabbe S., Krajnak J., Peyerl P., Sachs J., Thoma R. Detection and Localization of Persons Behind Obstacles Using M-Sequence Through-The-Wall Radar.

[30] Wren C., Azarbayejani A., Darrell T., Pentland A. (1997). Pfinder: Real-time tracking of the human body. IEEE Trans. Pattern Anal. Mach. Intell..

[31] Stauffer C., Grimson W. (2000). Learning patterns of activity using real-time tracking. IEEE Trans. Pattern Anal. Mach. Intell..

[32] Nag S., Barnes M. A Moving Target Detection Filter for an Ultra-Wideband Radar.

[33] Nag S., Fluhler H., Barnes M. Preliminary Interferometric Images of Moving Targets Obtained Using a Time-Modulated Ultra-Wide Band Through-Wall Penetration Radar.

[34] Toyama K., Krumm J., Brumitt B., Meyers B. Wallflower: Principles and Practice of Background Maintenance.

[35] Tipping M.E., Bishop C.M. (1999). Mixtures of probabilistic principal component analysers. Neural Comput..

[36] Poor H. (1994). An Introduction to Signal Detection and Estimation.

[37] Taylor J.D. (2001). Ultra-Wideband Radar Technology.

[38] Immoreev I., Fedotov D. Detection of UWB Signals Reflected from Complex Targets.

[39] Minkler G., Minkler J. (1990). CFAR: The Principles of Automatic Radar Detection in Clutter.

[40] Rohling H. (1983). Radar CFAR thresholding in clutter and multiple target situations. IEEE Trans. Aerosp. Electron. Syst..

[41] Dutta P., Arora A., Bibyk S. Towards Radar-Enabled Sensor Networks.

[42] Rovňaková J., Kocur D. (2010). TOA estimation and data association for through wall tracking of moving targets. EURASIP J. Wirel. Commun. Netw..

[43] Shen G., Zetik R., Yan H., Hirsch O., Thoma R.S. Time of Arrival Estimation for Range-Based Localization in UWB Sensor Networks.

[44] Rovňaková J., Kocur D. (2009). Compensation of wall effect for through wall tracking of moving targets. Radioeng. J..

[45] Aftanas M., Rovňaková J., Drutarovský M., Kocur D. Efficient Method of TOA Estimation for Through Wall Imaging by UWB Radar.

[46] Rovňaková J., Kocur D., Kažimír P. (2013). Investigation of localization accuracy for UWB radar operating in complex environment. Acta Polytech. Hung..

[47] Brookner E. (1998). Tracking and Kalman Filtering Made Easy.

[48] Brown R.G. (1983). Introduction to Random Signal Analysis and Kalman Filtering.

[49] Grewal M.S., Andrews A.P. (2003). Kalman Filtering: Theory and Practice.

[50] Arulampalam M.S., Maskell S., Gordon N., Clapp T. (2002). A tutorial on particle filters for online nonlinear/non-gaussian gayesian tracking. IEEE Trans. Signal Process..

[51] Nordlund P.-J., Gunnarsson F., Gustafsson F. Particle Filters for Positioning in Wireless Networks.

[52] Kolawole M. (2003). Radar Systems, Peak Detection and Tracking.

[53] Blackman S.S., Popoli R. (1993). Design and Analysis of Modern Tracking Systems.

[54] Chang S., Sharan R., Wolf M., Mitsumoto N., Burdick J.W. (2010). People tracking with UWB radar using a Multiple-Hypothesis Tracking of Clusters (MHTC) method. Int. J. Soc. Robot..

[55] Yu K., Montilleta J., Rabbachin A., Cheonga P., Oppermann I. (2006). UWB location and tracking for wireless embedded networks. Signal Process..

[56] Paolini E., Giorgetti A., Chiani M., Minutolo R., Montanari M. (2008). Localization capability of cooperative anti-intruder radar systems. EURASIP J. Adv. Signal Process..

[57] Eberly D. Intersection of Ellipses. Geometric Tools, LLC 1998–2008.

[58] Zetik R. (2011). Synchronization and Interference of M-sequence UWB Radar Systems. Personal Communication.

[59] Aftanas M., Rovňáková J., Rišková M., Kocur D., Drutarovský M. An Analysis of 2D Target Positioning Accuracy for M-Sequence UWB Radar System under Ideal Conditions.

[60] Yarovoy A., Matuzas J., Levitas B., Ligthart L. (2006). UWB radar for human being detection. IEEE Aerosp. Electron. Syst. Mag..

[61] Nezirovič A., Yarovoy A.G., Ligthart L.P. (2010). Signal processing for improved detection of trapped victims using UWB radar. IEEE Trans. Geosc. Remote Sens..

[62] Lv H., Lu G.H., Jing X.J., Wang J.Q. (2010). A new ultra-wideband radar for detecting survivors buried under earthquake rubbles. Microw. Opt. Technol. Lett..

